# Genetic insights into repurposing statins for hyperthyroidism prevention: a drug-target Mendelian randomization study

**DOI:** 10.3389/fendo.2024.1331031

**Published:** 2024-02-15

**Authors:** Anqi Huang, Xinyi Wu, Jiaqi Lin, Chiju Wei, Wencan Xu

**Affiliations:** ^1^ Department of Endocrinology and Metabolism, The First Affiliated Hospital of Shantou University Medical College, Shantou, China; ^2^ Shantou University Medical College, Shantou, China; ^3^ Multidisciplinary Research Center, Shantou University, Shantou, China

**Keywords:** thyroid, hyperthyroidism, statin, 3-hydroxy-3-methylglutarylcoenzyme reductase, blood lipids, drug target, genetics, Mendelian randomization

## Abstract

**Background:**

Current therapeutic measures for thyroid dysfunction are limited and often accompanied by adverse effects. The use of lipid-lowering drugs like statins has recently been associated with lower thyroid eye diseases risk.

**Objective:**

To investigate the implications of genetically proxied lipid-lowering drugs on thyroid dysfunction.

**Methods:**

In this drug-target Mendelian randomization (MR) study, we utilized genetic variants within drug target genes associated with low-density lipoprotein (LDL) or triglyceride (TG), derived from a genome-wide association study (GWAS) meta-analysis (N ≤ 188,577), to simulate lifelong drug interventions. Genetic summary statistics for thyroid dysfunction outcomes were retrieved from GWAS datasets of Thyroid Omics Consortium (N ≤ 54,288) and UK Biobank (N = 484,598). Inverse-variance-weighted MR (IVW-MR) method was performed as primary analysis, followed by validation in colocalization analysis. A subsequent two-step MR analysis was conducted to identify biomarkers mediating the identified drug-outcome association.

**Results:**

In IVW-MR analysis, genetic mimicry of 3-hydroxy-3-methylglutarylcoenzyme reductase (HMGCR) inhibitors (e.g. statins) was significantly associated with lower risk of hyperthyroidism in two independent datasets (OR_1_, 0.417 per 1-mmol/L lower in LDL-C; 95% CI 0.262 to 0.664; P_1 = _2.262 × 10^-4^; OR_2_ 0.996; 95% CI 0.993-0.998; P_2 = _0.002). Two-step MR analysis revealed eighteen biomarkers linked to genetic mimicry of HMGCR inhibition, and identified insulin-like growth factor 1 (IGF-1) levels mediating 2.108% of the negative causal relationship between HMGCR inhibition and hyperthyroidism.

**Conclusion:**

This study supports HMGCR inhibition as a promising therapeutic strategy for hyperthyroidism and suggests its underlying mechanisms may extend beyond lipid metabolism. Further investigations through laboratory studies and clinical trials are necessary to confirm and elucidate these findings.

## 1 Introduction

Thyroid dysfunction, encompassing hypothyroidism and hyperthyroidism, manifests as a prevalent health concern and an endocrine system disorder observed among adults, especially with a higher prevalence among women (1). This condition is characterized by deviations in the levels of serum thyroid stimulating hormone (TSH) and can manifest as overt or subclinical forms, characterized by abnormal TSH levels with or without accompanying symptoms, as well as abnormal and normal free thyroxine (FT4) levels, respectively. Notably, dyslipidemia is widely recognized to be frequently encountered in individuals with thyroid dysfunction, indicating an inherent association between thyroid hormone and lipid metabolism (2–4). Hyperthyroidism is characterized by lower levels of low-density lipoprotein cholesterol (LDL-C), triglycerides (TG), and total cholesterol (TC), alongside higher levels of high-density lipoprotein cholesterol (2, 5). On the other hand, both overt and subclinical hypothyroidism are linked to elevated TC and LDL-C levels. Similarly, TSH levels are observed to be connected to higher levels of TC, LDL-C, and TG, while free thyroxine has been shown to reduce cholesterol levels (2, 4, 6).

Studies have provided evidence supporting the significant impact of thyroid hormone on the heart and cardiovascular system (7). Some commonly observed manifestations of thyroid dysfunction have long been recognized as the results from the physiological effects of thyroid hormone on the cardiovascular system, including resting heart rate, left ventricular systolic force, atherosclerosis, systemic vascular resistance, and blood volume. In the management of cardiovascular disease, lipid-lowering drugs, such as statins, are commonly recommended for prescription with the aim of regulating atherosclerosis (8, 9). Given the established relationship between thyroid dysfunction and cardiovascular disease, it not uncommon for application of lipid-lowering perturbation in patients with thyroid-related cardiovascular disease.

However, the risks and benefits associated with lipid-lowering drugs in patients with thyroid dysfunction remain uncertain. A series of retrospective cohort studies and clinical trials have demonstrated that utilization of statin medication correlates with the restoration of thyroid function, a diminished occurrence of thyroid nodules, reduced thyroid volumes, and decreased thyroid autoimmunity in dyslipidemic patients, but the thyroid statuses of the study populations included in these investigations were varied (10–13). Conversely, a recent meta-analysis involving a large cohort of over 4 million subjects treated with statins and another retrospective analysis reported that hypothyroidism is a risk factor for statin intolerance (14, 15).

Randomized controlled clinical trials (RCTs) encounter challenges when studying thyroid-related diseases, primarily due to the requirement for adequate sample size and long-term follow-up (16). Consequently, it is imperative to investigate the potential causal link between lipid-lowering drugs and thyroid dysfunction through a robust approach that can provide valuable insights into the potential clinical effects of these drugs. Mendelian randomization (MR) emerges as a novel approach in this context, utilizing naturally randomized genetic variation in the gene encoding the protein drug target to effectively minimize the influence of unnoticed confounding factors, address potential reverse causality, and ultimately provide evidence comparable to that of RCTs (17). By utilizing genomic profile of individual patient, MR enables us to select medications that are more likely to improve outcomes in certain populations, while considering the side effects and adverse outcomes (18).

In the present study, our aim was to examine the impacts of perturbing four genetically proxied lipid-lowering drug targets on thyroid dysfunction outcomes (i.e. hyperthyroidism and hypothyroidism) and thyroid function traits (i.e. TSH and FT4). Therefore, a two-sample Mendelian randomization (MR) study was conducted utilizing openly accessible summary data from genome-wide association studies (GWAS) within European populations.

## 2 Materials and methods

### 2.1 Study design and ethics

**Figure 1:**
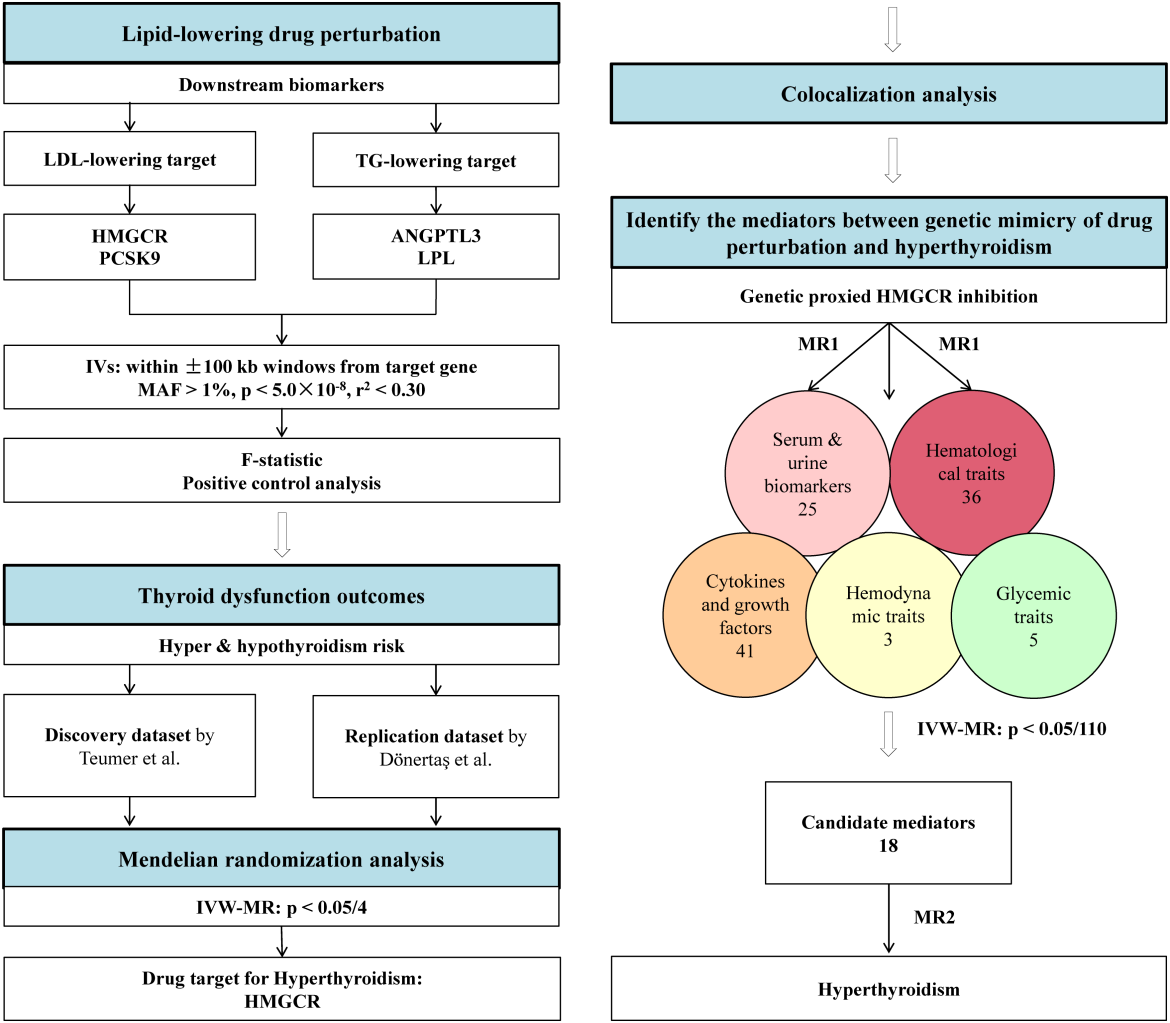
Outline for the Mendelian randomization study design. ANGPTL3, angiopoietin-like 3; HMGCR, 3-hydroxy-3-methylglutaryl CoA reductase; GWAS, genome-wide association study; IV, instrumental variable; IVW-MR, inverse-variance-weighted Mendelian randomization; LDL-C, low-density lipoprotein cholesterol; LPL, lipoprotein lipase; PCSK9, proprotein convertase subtilisin/kexin type 9; MAF, minor allele frequency; TG, triglyceride.

The outline of this study design is illustrated in **Figure 1**. First, we gained instrumental variables as genetic proxies for the therapeutic effect of four lipid-lowering drugs. Second, a drug-target MR was conducted to assess the causal impacts of drug perturbation on hype and hypothyroidism. Third, we performed colocalization analyses to validate the robustness of the significant MR results. Finally, we evaluated the MR associations of the identified target, HMGCR, with five categories of biomarkers, and subsequently identified the mediators to explore the possible underlying mechanisms from HMGCR inhibition to hyperthyroidism.

The ethical approvals of the utilized GWAS studies were obtained from the respective ethical review committees, and the ethics statement can be found in the original publications. Detailed information regarding the data source of this study can be obtained in **Supplementary Table S1**. This study was reported in reference to Strengthening the Reporting of Observational Studies in Epidemiology Using MR guidelines (19) (**Supplementary Table S2**).

### 2.2 Genetic proxies of lipid-lowering drugs

Summary-level genetic statistics of LDL-C and TG originated from the GWAS meta-analysis of lipid traits performed by the Global Lipids Genetics Consortium (GLGC) (20), which covered 1.7 million European participants. Genetic correlation was calculated through linear regression, adjusting for age, genomic principal components, and sex within each individual study, followed by meta-analysis across all included studies.

In this MR analysis, two licensed lipid-lowering drug classes, 3-hydroxy-3-methylglutaryl-coenzyme A reductase (HMGCR) inhibitors and proprotein convertase subtilisin/kexin type 9 (PCSK9) inhibitors, were included due to their primary pharmacological action of reducing LDL-C levels. For drug targets primarily involved in reducing TG, angiopoietin-like 3 (ANGPTL3) and lipoprotein lipase (LPL) were selected, considering their status as licensed or investigational therapeutics. Information regarding the pharmacological targets, mechanisms of action and corresponding encoding genes was obtained from the DrugBank database (https://go.drugbank.com/). Based on the predominant lipid-lowering effects of these drug targets that were identified in previous reviews (21–23) and the DrugBank database, the lipid-lowering effects of HMGCR and PCSK9 inhibition were assessed using genetically proxied LDL-C-lowering variants, while the lipid-lowering effects of ANGPTL3 inhibition and LPL activation were examined using genetically proxied TG-lowering variants.

Referring to similar methods in previous drug-target MR studies (24–26), in inverse variance-weighted Mendelian randomization (IVW-MR) analysis, cis-variants were chosen as proxies for the therapeutic effects of HMGCR, PCSK9, ANGPTL3 inhibition, and LPL activation, meeting the following criteria: (i) significant association with the pertinent lipid traits while presenting no association with the corresponding outcome (at P<5×10^−8^); (ii) location within ±100 kb of the target gene; (iii) limited linkage disequilibrium (r^2^< 0.3) with other variants within the same region, in reference to PLINK software (27) and phase 3 of 1000 genomes data (28), to avoid multicollinearity; (iv) strand-correction for non-palindromic SNPs and exclusion of palindromic ones, to avoid ambiguity in identifying the effect alleles.

### 2.3 Data source for thyroid dysfunction and thyroid function traits

We obtained summary-level genetic data for thyroid dysfunction from two independent GWASs. A meta-analysis of GWASs in European population conducted by Thyroid Omics Consortium was selected as the discovery dataset (29). This GWAS meta-analysis comprised a substantial sample size, including up to 1,840 hyperthyroidism cases, 3,440 hypothyroidism cases, and 49,983 controls with TSH levels within the reference range. Hyper- and hypothyroidism were clearly defined by TSH levels that deviated below and above the cohort-specific reference range (29), respectively. Overt and mild subclinical hyper- and hypothyroidism patients were included. Participants who disclosed the usage of thyroid medication or had a past record of thyroid surgery were not included in the study cohort. Genetic statistics for TSH and FT4 were also obtained from this GWAS meta-analysis. No sample overlap was reported between the GWAS of the GLGC and the Thyroid Omics Consortium.

For the replication analyses, data were extracted from an additional GWAS dataset of 484,598 UK Biobank participants aged 0 to 70 years (30). The GWAS comprised 3,731 hyperthyroidism cases and 23,497 hypothyroidism cases. Cases were defined based on a self-reported disease hierarchical structure, whereby hyperthyroidism was defined as *hyperthyroidism or thyrotoxicosis* (code 1225 in field 20002), and hypothyroidism was defined as *hypothyroidism or myxedema* (code 1226 in field 20002). Participants entered self-reported disease information validated by interview at baseline with a trained nurse. Although the sample size and number of cases in this dataset were relatively larger compared to the discovery dataset, the definition criteria in this dataset were relatively less precise. This GWAS was performed using linear mixed models with adjustments for potential confounders such as sex, age, body mass index (BMI), and other relevant factors as covariates in the analysis. The methods employed for genotyping and quality control in the UK Biobank study have been previously outlined (31).

### 2.4 IVW-MR analysis

We applied the multiplicative random-effects IVW-MR method to estimate the effects of genetically proxied inhibitors of HMG-CoA, PCSK9, ANGPTL3 and activators of LPL on hyper- and hypothyroidism (32). Estimates of MR were reported as odds ratios (OR) that corresponded to a deviation in the risk for the thyroid dysfunction endpoint per 1-SD decrease in the respective lipid fraction.

The validity of causal estimation in MR study hinges upon the satisfaction of three key assumptions (33). First, the genetic instrumental variables (IVs) should exhibit a strong association with the exposure of interest. Second, genetic IVs affect the outcome only through their effect on the exposure. Third, genetic IVs are independent of confounding factors that influence the outcome of interest.

We estimated the F statistic for each drug target IV to mitigate the potential for weak instrument bias. Calculated by the formula:


$$F=beta^{2}/se^{2}$$

IV with F-statistic exceeding 10 was considered to be indicative of sufficient IV strength for analysis (34). Moreover, the statistical power for the MR analysis was computed utilizing an online tool, namely mRnd (https://shiny.cnsgenomics.com/mRnd/) (35). To further examine the validity of the IVs for drug targets, positive control analyses were conducted utilizing coronary artery disease (CAD) as the positive control outcome, based on the established therapeutic effect of lipid-lowering therapy in this context. Summary-level data for CAD were procured from the CARDIo-GRAMplusC4D dataset (predominantly European ancestry, 60,801 cases, 123,504 controls) (36).

To address potential confounding and evaluate the robustness of MR results, we employed a variety of sensitivity analyses. First, to assess the heterogeneity within IVs, the Cochran’s Q test was performed with a significance threshold of p< 0.05 (32). Second, to account for directional horizontal pleiotropy, the MR-Egger regression method was applied, of which intercept term at p< 0.05 indicates an estimate of the directional pleiotropic effect (32). Third, the weighted median and mode methods were performed to further examine the robustness of the inverse variance–weighted estimates to uncorrelated horizontal pleiotropy (37). Finally, leave-one-out analyses were employed to assess the impact of single SNPs in drug-target proxies on the overall causal estimates (38).

Additionally, we also investigated the impact of genetically proxied lipid-lowering drugs on thyroid hormones to further examine observed findings on thyroid dysfunction outcomes.

Bonferroni-corrected significance levels of P-value< 0.0125 (4 drug targets, 0.05/4) were utilized to adjust for multiple testing of MR. Analyses of MR were performed in R program (v4.2.2) using the *TwoSampleMR* (v0.5.6) (39).

### 2.5 Bayesian colocalization analysis

For further examination of the drug targets that exhibited statistical significance for the thyroid dysfunction risk among MR analysis, Bayesian colocalization analysis was employed as an extension sensitivity analysis of MR. By using the coloc method with default priors setting (40), it enabled assessment for whether identified associations in MR were due to linkage disequilibrium, and provided the posterior probability for five hypotheses on whether an individual variant is shared between two traits. A posterior probability of hypothesis 4 (PP.H4) exceeding 80% signifies the potential presence of a shared causal variant affecting both the expression of the target gene and the risk of thyroid dysfunction. The R package *coloc* (v5.2.1) was used for Bayesian colocalization analyses (40).

### 2.6 Lipid traits, the risk of thyroid dysfunction outcomes and thyroid function traits

Considering the existing evidence linking lipids to thyroid function and thyroid diseases, as documented in prior studies (2, 3, 5), to investigate whether the association between genetic proxies of lipid-lowering drugs and thyroid dysfunction risk was independent of lipid-lowering effect, we additionally assessed the potential association between LDL-C and TG levels and the risk of thyroid dysfunction, as well as their association with thyroid function traits by using genome-wide significant variants characterized by low pairwise correlations (threshold r^2^< 0.001).

### 2.7 Biomarker-wide association and mediation analysis

Furthermore, we conducted a two-step MR analysis to identify mediator variables mediating the causal relationship between genetic mimicry of drug perturbation and thyroid dysfunction outcomes. Firstly, we evaluated the MR association of identified genetically proxied drug perturbations with five common biomarker categories, covering serum and urine biochemical (41), hematological (42), inflammatory (43), hemodynamic (44) and glycemic traits (45, 46) (**Supplementary Table S1**). Bonferroni-corrected significance levels of P-value< 4.55E^-04^ (0.05/110) were used to adjust for multiple testing. Subsequent mediation analyses were performed to explore the pathophysiological pathways underlying the identified drug-outcome association. The details of the GWAS data source for these biomarkers are provided in the **Supplementary Table S1**. In this two-step MR analysis, the direct effect of exposure on outcome was measured by β0 − β1 × β2 (47), wherein β0 represents the causal impact of the exposure on the outcome, β1 denotes the causal effect of the exposure on the mediator, β2 represents the causal effect of the mediator on the outcome, and β1 × β2 denotes the mediating effect from the exposure to the outcome. The assessment of indirect effects and mediated proportions was carried out using the “Product of coefficients” method, with standard errors for the indirect effects derived through the delta method (47).

## 3 Results

### 3.1 Genetic instruments selection for drug targets

**Figure 2:**
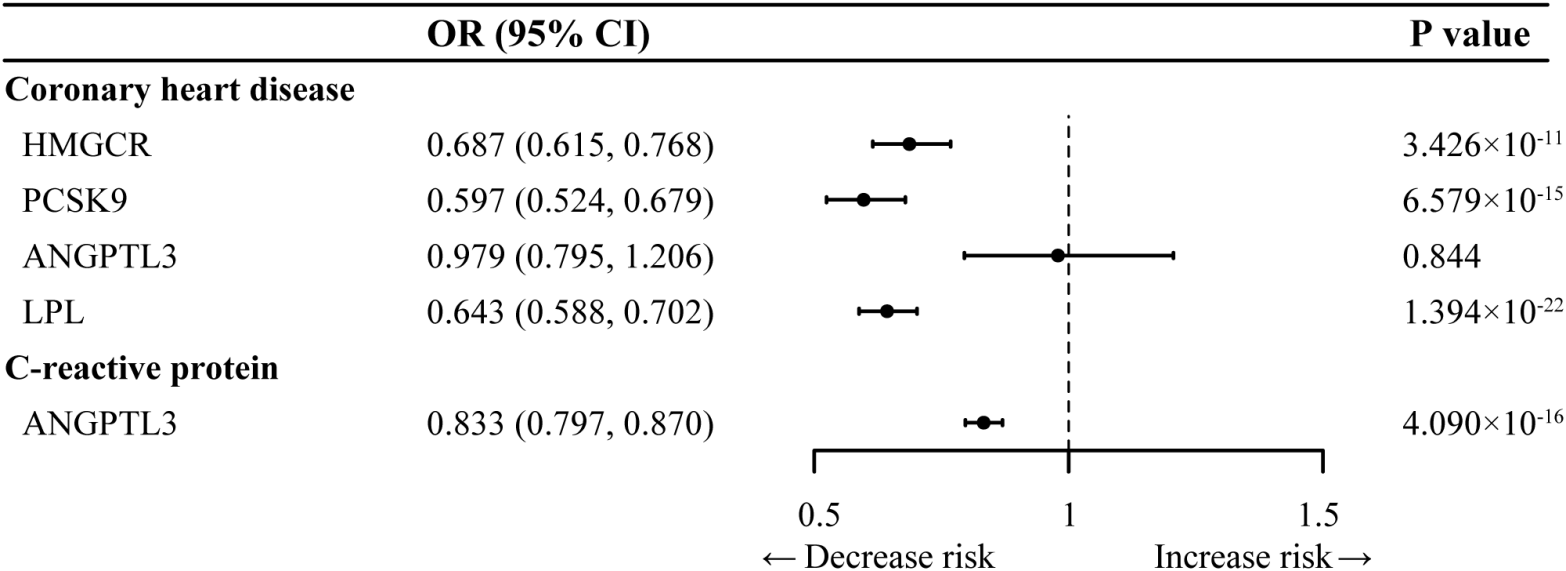
Positive control analysis for genetic instruments selected in Inverse-variance-weighted Mendelian randomization analysis. ANGPTL3, angiopoietin-like 3; HMGCR, 3-hydroxy-3-methylglutaryl CoA reductase; LPL, lipoprotein lipase; PCSK9, proprotein convertase subtilisin/kexin type 9; OR, odds ratio.

We selected 8 SNPs to proxy the LDL-C-lowering effect via HMGCR inhibition, 13 SNPs for LDL-C-lowering via PCSK9 inhibition, 5 SNPs for TG-lowering via ANGPTL3 inhibition, and 21 SNPs for TG-lowering via LPL activation (**Table 1**, **Supplementary Table S3**). All the genetic IVs exhibited F statistics exceeding 30, indicating a low likelihood of the results being influenced by weak instrument bias. Furthermore, as demonstrated in positive control analyses, almost all genetically proxied drug targets exhibited significant associations with a reduced risk of coronary artery disease (**Figure 2**), ensuring the validity of the used IVs. Regarding ANGPTL3, which did not align with the above pattern but was consistent with previous MR studies (23, 48), we therefore conducted additional analysis into the association of genetically proxied ANGPTL3 inhibition with a previously reported pertinent biomarker, C-reactive protein (48), and observed a statistically significant outcome.

**Table 1:** Summary information of lipid-lowering drug and corresponding targets.

Primary pharmacological action	Drug target	Drug class	Drug example	Encoding gene^a^	Gene region^b^	nSNP	Mean F
Reduced LDL-C	HMG-CoA reductase	HMG-CoA reductase inhibitors	Statins	*HMGCR*	chr5:74632993-74657941	8	149
	Proprotein convertase subtilisin/kexin type 9	Proprotein convertase subtilisin/kexin type 9 inhibitors	Evolocumab	*PCSK9*	chr1:55505221-55530525	13	167
Reduced TG	Angiopoietin-like protein 3	Angiopoietin- like protein 3 inhibitors	Evinacumab	*ANGPTL3*	chr1:63063191-63071984	5	192
	Lipoprotein lipase	Investigational target	NA	*LPL*	chr8:19796764-19824770	21	209

^a^Genes that encode pharmacologic targets of these drugs were identified utilizing the DrugBank database (https://go.drugbank.com/). ^b^Based on GRCh37/hg19 by NCBI. LDL-C, low-density lipoprotein cholesterol; SNPs, single nucleotide polymorphisms; TG, triglyceride.

### 3.2 Lipid-lowering drug targets and the risk of thyroid dysfunction outcomes

**Figure 3:**
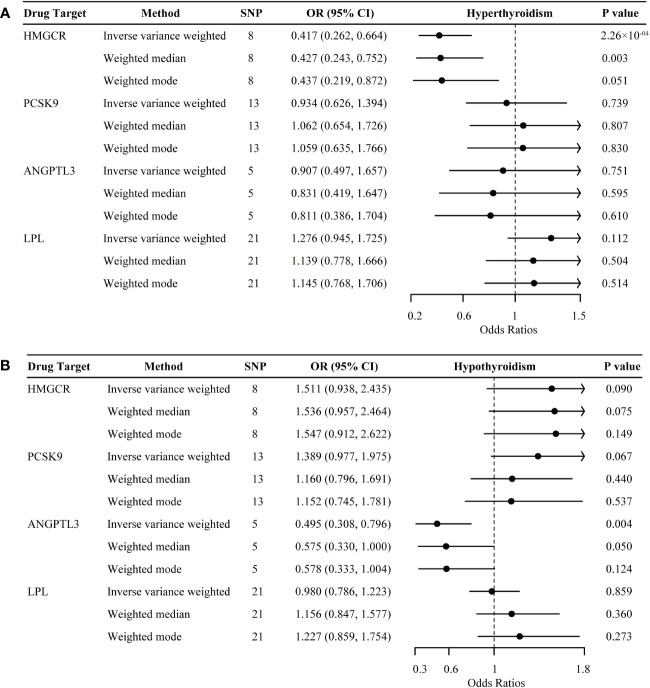
Inverse-variance-weighted Mendelian randomization association of genetically proxied lipid-lowering drugs with the risk of hyperthyroidism **(A)** and hypothyroidism **(B)**. Data are represented as odds ratios (ORs) with 95% confidence intervals (error bars). An OR less than 1.000 indicates a decreased risk of disease associated with lipid-lowering drug intervention. The associations were considered significant after correcting for multiple testing (p< 0.05/4). ANGPTL3, angiopoietin-like 3; HMGCR, 3-hydroxy-3-methylglutaryl CoA reductase; LPL, lipoprotein lipase; PCSK9, proprotein convertase subtilisin/kexin type 9.

For hyperthyroidism, as shown in the IVW-MR analysis, genetically proxied inhibition of HMGCR was significantly associated with a decreased risk in both the discovery dataset (OR, 0.417 per 1-mmol/L lower in LDL-C; 95% CI 0.262 to 0.664; P = 2.262 × 10^-4^) (**Figure 3A**) and the replication dataset (OR, 0.996; 95% CI 0.993 to 0.998; P = 0.002) (**Supplementary Table S4**). While no other significant results were observed in the discovery dataset for hyperthyroidism, the replication dataset indicated additional significant associations, including genetically proxied ANGPTL3 inhibition linked to a reduced risk of hyperthyroidism (OR, 0.995 per 1-mmol/L lower in TG; 95% CI 0.992 to 0.998; P = 0.002), and genetically proxied LPL activation associated with an increased risk of hyperthyroidism (OR, 1.003; 95% CI 1.001 to 1.005; P = 4.150 × 10^-4^). However, IVW-MR analyses did not demonstrate any significant link between genetically mimicry of PCSK9 inhibition and hyperthyroidism risk.

For hypothyroidism, genetically proxied ANGPTL3 inhibition demonstrated significant associations with a reduced risk in the discovery dataset (OR, 0.495 per 1-mmol/L lower in TG; 95% CI 0.308 to 0.796; P = 0.004) (**Figure 3B**), but this association did not replicate in the independent replication dataset (OR, 1.002; 95% CI 0.994 to 1.010; P = 0.638) (**Supplementary Table S5**). In the replication dataset, there was a trend towards an increased risk of hypothyroidism for genetically proxied inhibition of HMGCR (OR, 1.008 per 1-mmol/L lower in LDL-C; 95% CI 1.001 to 1.014; P = 0.019) and a trend in a decreased risk of hypothyroidism for genetically proxied inhibition PCSK9 (OR, 0.995; 95% CI 0.990 to 1.000; P = 0.031). However, these tendencies did not survive after multiple-testing adjustments. In addition, genetic mimicry of LPL activation exhibited neutral effects on hypothyroidism risk in both datasets.

The alternative MR analyses were generally in line with the results in IVW-MR (**Supplementary Tables S4**, **S5**). Sensitivity analyses did not reveal any significant evidence of heterogeneity in the Cochran’s Q test (**Supplementary Table S6**), bias from horizontal pleiotropy in MR-Egger intercept (**Supplementary Table S6**), or substantial influence of any outlier SNP on the overall estimate in the leave-one-out analyses (**Supplementary Table S7**) for the above results.

### 3.3 Lipid-lowering drug targets and thyroid function traits

**Figure 4:**
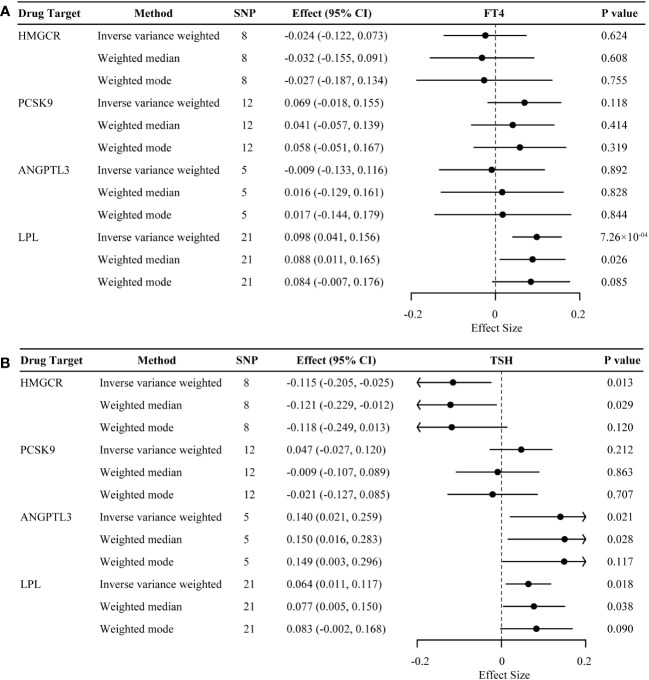
Inverse-variance-weighted Mendelian randomization association of genetically proxied lipid-lowering drugs with levels of free thyroxine (FT4) **(A)** and thyroid stimulating hormone (TSH) **(B)**. Data are represented as effect sizes with 95% confidence intervals (error bars). An effect size less than 0.000 indicates a decreased effect of the trait associated with lipid-lowering drug intervention. The associations were considered significant after correcting for multiple testing (p< 0.05/4). ANGPTL3, angiopoietin-like 3; HMGCR, 3-hydroxy-3-methylglutaryl CoA reductase; LPL, lipoprotein lipase; PCSK9, proprotein convertase subtilisin/kexin type 9.

As illustrated in **Figure 4**, genetic mimicry of LPL activation was significantly associated with the increased level of FT4 within reference range (β = 0.098; 95% CI 0.041 to 0.156; P = 0.001), partially supporting the observed detrimental effect of genetically proxied LPL activation on hyperthyroidism risk above. Suggestive evidence demonstrated a decreased tendency of TSH within reference range for genetic mimicry of HMGCR inhibition (β = -0.115; 95% CI -0.205 to -0.025; P = 0.013), and an increased tendency of TSH within reference range for genetic mimicry of LPL activation (β = 0.064; 95% CI 0.011 to 0.117; P = 0.018) and ANGPTL3 inhibition (β = 0.140; 95% CI 0.021 to 0.259; P = 0.021). Genetically proxied PCSK9 inhibition demonstrated neutral effects on thyroid function, which was consistent with the above results.

### 3.4 Colocalization analysis of genetically proxied HMGCR inhibition with hyperthyroidism

Bayesian colocalization analyses were further conducted to assess the probability of sharing the same causal variant within the given locus between genetically proxied inhibition of HMGCR and hyperthyroidism (**Supplementary Table S8**). Although the posterior probability of shared causal variants within ± 500kb windows of the *HMGCR* gene region (PP.H4 = 7.576% in discovery dataset; PP.H4 = 0.022% in replication dataset) was low, the probability of colocalization conditional on the presence of a causal variant for hyperthyroidism [PP.H4/(PP.H3 + PP.H4)] exceeded 75% (82.980% in discovery dataset; 75.124% in replication dataset), providing suggestive evidence for colocalization.

### 3.5 Lipid traits were not associated with thyroid dysfunction risk or thyroid function

In order to determine whether the impacts of lipid-lowering drug targets on the studied outcomes were mediated by their lipid-lowering effects, we conducted additional IVW-MR analyses to assess the association between lipid traits and the above thyroid-related outcomes. A total of 75 independent SNPs associated with LDL-C and 53 SNPs associated with TG were identified as IVs to genetically proxy the reduction in lipid traits (**Supplementary Tables S9**, **S10**). Neither IVW-MR nor any alternative MR method revealed any causal effect of LDL-C or TG on the risk of thyroid dysfunction outcomes or thyroid function traits (**Supplementary Tables S11**-**S13**).

### 3.6 Causal association of genetically proxied HMGCR inhibition with multiple categories of biomarkers

**Figure 5:**
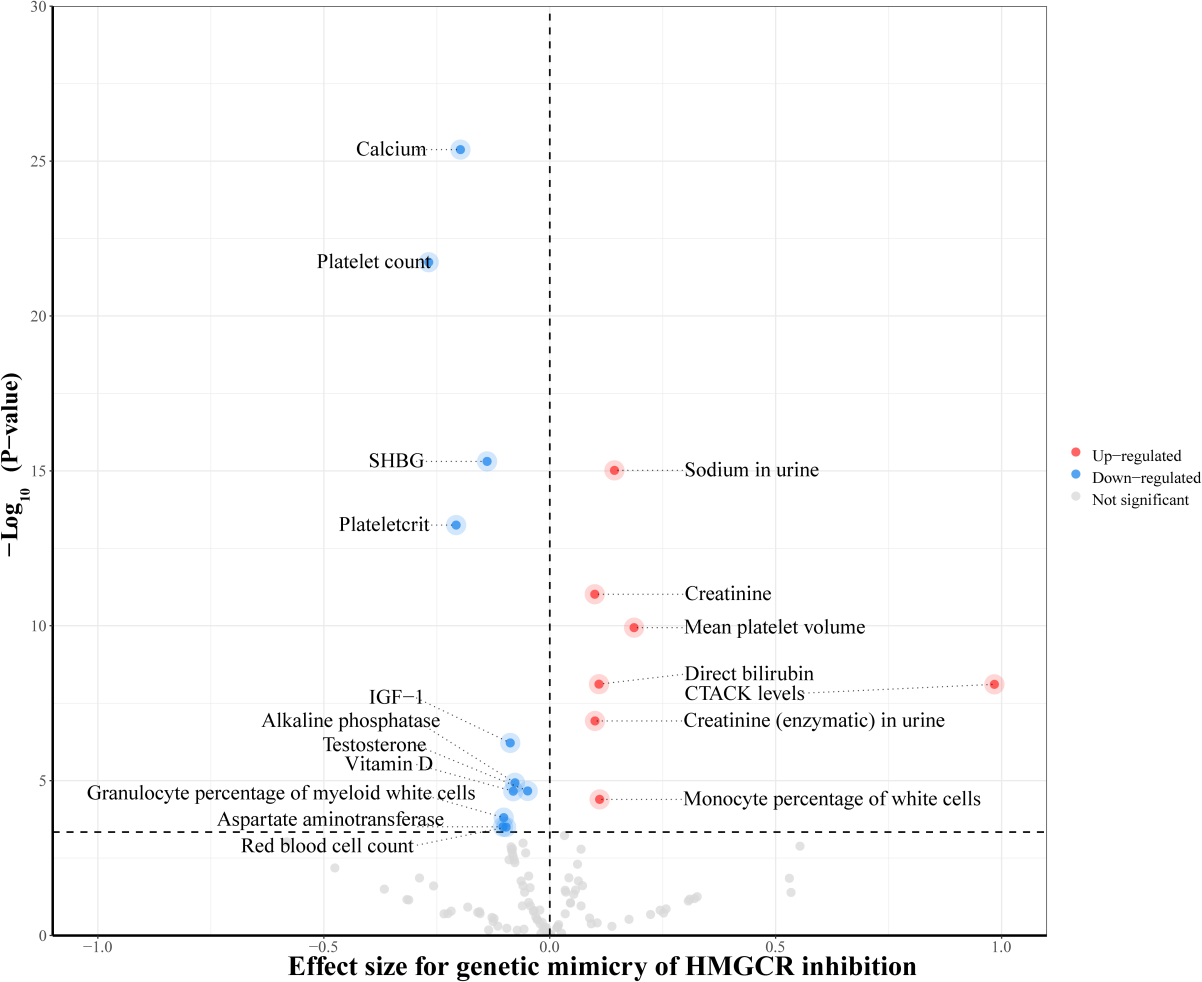
Inverse-variance-weighted Mendelian randomization association of genetically proxied 3-hydroxy-3-methylglutaryl CoA reductase (HMGCR) inhibition with levels of 110 biomarkers. The drug-biomarker associations survived after multiple testing were colored in the plot. CTACK, cutaneous T-cell attracting chemokine; IGF-1, insulin-like growth factor 1; SHBG, sex hormone-binding globulin.

**Figure 6:**
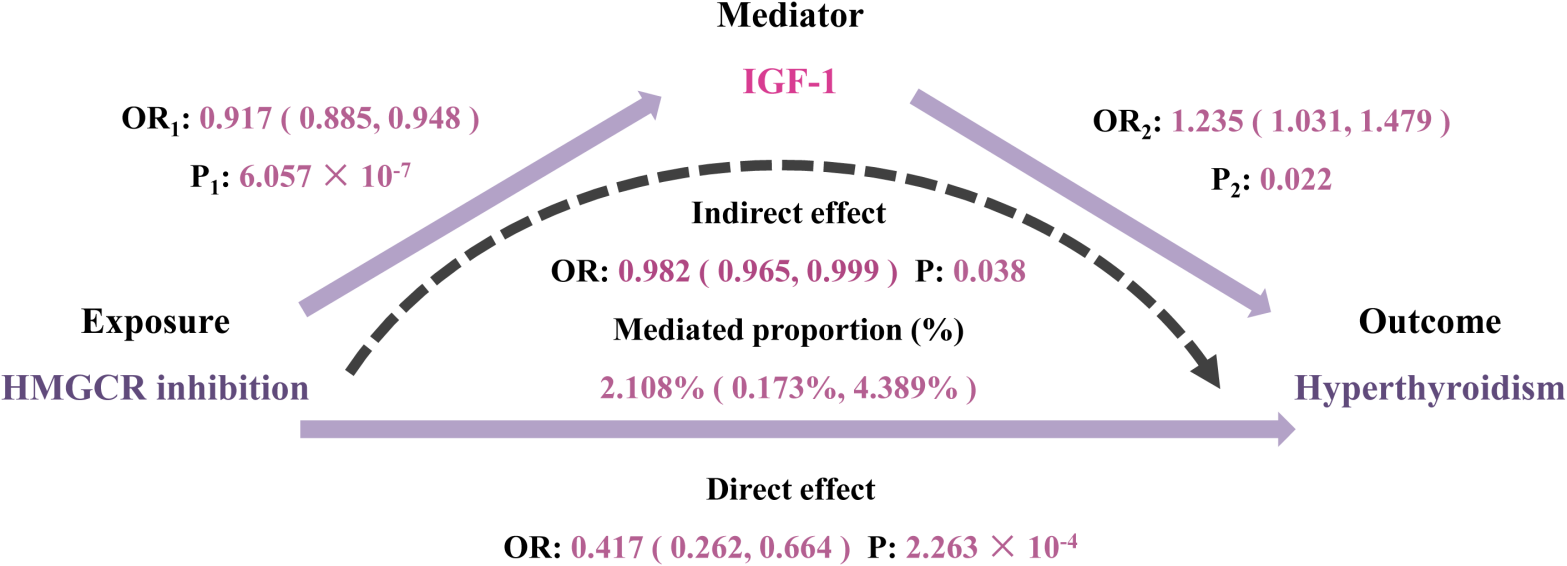
Two-step Mendelian randomization analysis of the effect of 3-hydroxy-3-methylglutaryl CoA reductase (HMGCR) inhibition on hyperthyroidism via potential mediators. IGF-1, insulin-like growth factor 1; OR, odds ratio.

To investigate the underlying mechanisms of the identified drug target, HMGCR, in relation to hyperthyroidism, we further utilized two-step MR method. We extended to explore the relationship between genetically proxied HMGCR inhibition and 110 biomarkers classified into 5 categories. Detailed information regarding the data sources for the biomarkers is listed in **Supplementary Table S11**. After multiple-testing adjustment, we found genetic mimicry of HMGCR inhibition was associated with elevated levels of 7 biomarkers and reduced levels of 11 biomarkers, mainly in serum biochemistry and hematological traits (**Figure 5**, **Supplementary Table S14**). In the second step, among these 18 biomarkers, we observed IGF-1 exhibiting a significant mediating role between HMGCR inhibition and hyperthyroidism risk (mediation proportion: 2.108%; 95% CI 0.173% to 4.389%; P = 0.038) (**Figure 6**, **Supplementary Table S15**).

## 4 Discussion

This study explored the causal relationships between four lipid-lowering drug targets and thyroid dysfunction risk. We found significant evidence supporting HMGCR inhibition as a promising therapeutic target for hyperthyroidism, validated across two independent datasets. Interestingly, lipid traits exhibited a neutral impact on all thyroid-related outcomes, and the remaining lipid-lowering drug targets did not present compelling evidence to be beneficial for hyperthyroidism. Taken together, our results suggest that the mechanism for the therapeutic effect of HMGCR inhibition on hyperthyroidism may be independent of its lipid-lowering action. Moreover, two-step MR analyses revealed that the risk-reducing effect of HMGCR inhibition on hyperthyroidism was partially mediated by lowering the levels of insulin-like growth factor 1 (IGF-1). Additionally, our study also provided suggestive evidence for the potential therapeutic use of ANGPTL3 inhibitors for both hypothyroidism and hyperthyroidism, while showing that LPL activation was related to increasing hyperthyroidism risk. To our knowledge, this is the first MR study to investigate the association of lipid-lowering drug targets with the risk of thyroid dysfunction.

Statins, a class of drugs predominantly targeting HMGCR, have emerged as potential candidates for the therapeutic intervention of Graves’ orbitopathy (GO), an autoimmune extrathyroidal manifestation in ~25-30% of Graves’ disease (GD) patients (49). A retrospective study by Stein et al. involving a large cohort of newly-diagnosed GD patients provided compelling evidence for the potential therapeutic effects of statins in preventing and treating GO (50). This observation was further supported by another recent retrospective study, in which statin intervention in newly diagnosed patients with GD may decrease the risk of GO (51). Moreover, Lanzolla et al. further conducted a phase 2 randomized clinical trial demonstrating the beneficial effects of atorvastatin, a statin, when added to an intravenous glucocorticoid regimen for GO in hypercholesterolemic patients with GD (52). Notably, beyond their association with GO, statins have also exhibited promise in reducing the risk of other autoimmune disorders, including rheumatoid arthritis (53) and multiple sclerosis (54). The cumulative evidence from these studies suggests that HMGCR, the primary target of statins, could serve as a therapeutic target for patients with hyperthyroidism. However, due to their limited sample size, this speculation needs to be validated in more extensive studies to determine its applicability in individuals with hyperthyroidism, irrespective of orbital involvement. Interestingly, our two-step MR analyses identified that IGF-1 served as a mediator for the protective effect of HMGCR inhibition on hyperthyroidism. This mediation effect provides a plausible mechanistic insight into the observed therapeutic effects in MR analyses, as it aligns with the overexpression of IGF-1 receptors (IGF-1R) observed in individuals with thyroid eye disease (TED) (55). An anti-IGF-1R antibody, teprotumumab, has recently been shown to be effective in attenuating various TED manifestations in a phase II clinical trial (56), further supporting the potential therapeutic significance of targeting HMGCR in hyperthyroidism.

Our study suggests that the protective action of HMGCR inhibitors on hyperthyroidism may be beyond their cholesterol-lowering properties. This speculation is compatible with the outcomes of studies conducted by Stein et al. and Nilsson et al., where non-statin lipid-lowering drugs did not show a significant impact on the development of Graves’ orbitopathy (50, 51). This protective action of statins on GO is considered to originate from their pleiotropic actions, anti-inflammatory effects (49, 57). Statins can function by inhibiting the mevalonate pathway, which induces a significant reduction of protein prenylation, subsequently affecting autophagic events and exerting a coregulatory effect on apoptosis and unfolded protein response stress (58). Autophagic and apoptosis programs, with their influence on the innate immune signaling pathway, play vital roles in maintaining the inflammation balance. Thus, through these mechanisms, statins have the potential to modulate innate immunity and regulate the balance of inflammatory cells. Additionally, *in-vitro* studies and experimental animal models of autoimmune diseases have provided evidence of statins’ direct immunoregulatory effects. They can induce tolerogenic dendritic cells, a specialized subset of dendritic cells, and subsequently promote immune tolerance and counteract autoimmune responses (59). Taken together, these findings raise the possibility that the protective mechanisms of statins for hyperthyroidism extend beyond lipid metabolism. Further research is necessary to uncover the precise mechanisms underlying the therapeutic impacts of statins and assess their clinical effectiveness and safety in hyperthyroidism patients, regardless of their serum lipid fraction.

We also found suggestive evidence supporting the potential therapeutic use of ANGPTL3 inhibitors for both hypothyroidism and hyperthyroidism. In contrast, LPL activation, known as the primary action of ANGPTL3 inhibitors (60), was found to be related to an increased risk of hyperthyroidism. This contradictory finding finds support in genetic studies that have demonstrated highly similar systemic metabolic effects for genetic proxies of ANGPTL4 inhibition and LPL activation, whereas inhibition of genetic mimicry of ANGPTL3 leads to distinct metabolic consequences (48, 61). Further studies are warranted to validate these findings.

The present MR study has several notable merits. Firstly, compared to conventional clinical and experimental studies, MR provides a more effective approach for discerning the lifelong adverse effects of genetically determined lipid-lowering drugs on thyroid dysfunction, as it reflects long-term impacts rather than short-term effects on lipid levels resulting from drug treatment. Secondly, we explored the association of lipid-lowering drug targets with the risk of thyroid dysfunction in two independent datasets for validation, enhancing the robustness of effect estimates and findings. Thirdly, by employing various methods to verify the efficacy of genetic instruments as proxies for the investigated drugs, this study minimized confounding factors and prevented reverse causation. Finally, the robustness was further strengthened through the implementation of multiple sensitivity analyses, which assessed the effectiveness of the instruments and tested the MR assumptions, ensuring more reliable causal inferences.

Several limitations should be considered. Firstly, the genetically predicted effects of drugs may not fully reflect their actual clinical therapeutic effects, potentially introducing bias. Secondly, despite leveraging two extensive GWAS datasets for hyperthyroidism, the limited number of hyperthyroidism cases underscores the need for replication analyses with a larger sample size and specific hyperthyroidism GWAS in the future. In addition, GWAS summary data for thyroid function traits may introduce potential survival bias that may limit the generalizability of our findings. Fourthly, while efforts were made to minimize confounding bias and address horizontal pleiotropy, the possibility of residual bias cannot be completely ruled out. Lastly, as the GWAS data utilized in this study is circumscribed to individuals of European ancestry, caution should be exercised in extending these findings to different populations.

## 5 Conclusion

Our study highlights HMGCR inhibition as a promising therapeutic target for hyperthyroidism, and reveals a potential mediation pathway involving the modulation of IGF-1 levels rather than lipid metabolism. Furthermore, the study suggests potential therapeutic implications of ANGPTL3 inhibition for both hypothyroidism and hyperthyroidism, alongside the observed risk increase in hyperthyroidism with LPL activation. These findings contribute novel insights to the understanding of lipid-lowering drug targets in thyroid dysfunction. Elucidating the underlying mechanisms of these findings warrants further investigation through laboratory studies and clinical trials.

## Data availability statement

The original contributions presented in the study are included in the article/**Supplementary Material**. Further inquiries can be directed to the corresponding author.

## Ethics statement

Ethical approval was not required for the present study as it relied solely on publicly available summary-level data. The ethical approvals of the original studies were obtained from the respective ethical review committees, and the ethics statement can be found in the original publications. Detailed information regarding the summary data used in this study can be found in **Supplementary Table S1**.

## Author contributions

AH: Conceptualization, Data curation, Formal analysis, Investigation, Methodology, Software, Writing – original draft. XW: Data curation, Software, Validation, Visualization, Writing – original draft. JL: Data curation, Software, Validation, Visualization, Writing – review & editing. CW: Conceptualization, Funding acquisition, Methodology, Project administration, Resources, Writing – review & editing. WX: Conceptualization, Funding acquisition, Methodology, Project administration, Resources, Supervision, Validation, Writing – review & editing.

## References

[1] Taylor PN Albrecht D Scholz A Gutierrez-Buey G Lazarus JH Dayan CM. Global epidemiology of hyperthyroidism and hypothyroidism. Nat Rev Endocrinol. (2018) 14:301–16. doi: 10.1038/nrendo.2018.18 29569622

[2] Duntas LH. Thyroid disease and lipids. Thyroid. (2002) 12:287–93. doi: 10.1089/10507250252949405 12034052

[3] van Vliet NA Bos MM Thesing CS Chaker L Pietzner M Houtman E. Higher thyrotropin leads to unfavorable lipid profile and somewhat higher cardiovascular disease risk: Evidence from multi-cohort Mendelian randomization and metabolomic profiling. BMC Med. (2021) 19:266. doi: 10.1186/s12916-021-02130-1 34727949 PMC8565073

[4] Su X Peng H Chen X Wu X Wang B. Hyperlipidemia and hypothyroidism. Clin Chim Acta. (2022) 527:61–70. doi: 10.1016/j.cca.2022.01.006 35038435

[5] Jung KY Ahn HY Han SK Park YJ Cho BY Moon MK. Association between thyroid function and lipid profiles, apolipoproteins, and high-density lipoprotein function. J Clin Lipidol. (2017) 11:1347–53. doi: 10.1016/j.jacl.2017.08.015 28958565

[6] Sinha RA Singh BK Yen PM. Direct effects of thyroid hormones on hepatic lipid metabolism. Nat Rev Endocrinol. (2018) 14:259–69. doi: 10.1038/nrendo.2018.10 PMC6013028 29472712

[7] Klein I Danzi S. Thyroid disease and the heart. Curr Probl Cardiol. (2016) 41:65–92. doi: 10.1016/j.cpcardiol.2015.04.002 26792255

[8] Grundy SM Stone NJ Bailey AL Beam C Birtcher KK Blumenthal RS. 2018 AHA/ACC/AACVPR/AAPA/ABC/ACPM/ADA/AGS/APhA/ASPC/NLA/PCNA guideline on the management of blood cholesterol: Executive summary: A report of the American College of cardiology/American heart association task force on clinical practice guidelines. J Am Coll Cardiol. (2019) 73:3168–209. doi: 10.1016/j.jacc.2018.11.002 30423391

[9] Kim K Ginsberg HN Choi SH. New, novel lipid-lowering agents for reducing cardiovascular risk: Beyond statins. Diabetes Metab J. (2022) 46:517–32. doi: 10.4093/dmj.2022.0198 PMC9353557 35929170

[10] Cappelli C Castellano M Pirola I De Martino E Gandossi E Delbarba A. Reduced thyroid volume and nodularity in dyslipidaemic patients on statin treatment. Clin Endocrinol (Oxf). (2008) 68:16–21. doi: 10.1111/j.1365-2265.2007.02982.x 17666091

[11] Wang Y Li Q Yuan Z Ma S Shao S Wu Y. Statin use and benefits of thyroid function: A retrospective cohort study. Front Endocrinol (Lausanne). (2021) 12:578909. doi: 10.3389/fendo.2021.578909 33737906 PMC7962670

[12] Krysiak R Kowalcze K Okopień B. The effect of statin therapy on thyroid autoimmunity in patients with Hashimoto’s thyroiditis: A pilot study. Pharmacol Rep. (2016) 68:429–33. doi: 10.1016/j.pharep.2015.11.005 26922549

[13] Gullu S Emral R Bastemir M Parkes AB Lazarus JH. *In vivo* and *in vitro* effects of statins on lymphocytes in patients with Hashimoto’s thyroiditis. Eur J Endocrinol. (2005) 153:41–8. doi: 10.1530/eje.1.01941 15994744

[14] Bytyçi I Penson PE Mikhailidis DP Wong ND Hernandez AV Sahebkar A. Prevalence of statin intolerance: a meta-analysis. Eur Heart J. (2022) 43:3213–23. doi: 10.1093/eurheartj/ehac015 PMC9757867 35169843

[15] Robison CD Bair TL Horne BD McCubrey RO Lappe DL Muhlestein JB. Hypothyroidism as a risk factor for statin intolerance. J Clin Lipidology. (2014) 8:401–7. doi: 10.1016/j.jacl.2014.05.005 25110221

[16] Medici M Peeters RP Teumer A Taylor P. The importance of high-quality mendelian randomisation studies for clinical thyroidology. Lancet Diabetes Endocrinol. (2019) 7:665–7. doi: 10.1016/S2213-8587(19)30145-7 31076377

[17] Gill D Georgakis MK Walker VM Schmidt AF Gkatzionis A Freitag DF. Mendelian randomization for studying the effects of perturbing drug targets. Wellcome Open Res. (2021) 6:16. doi: 10.12688/wellcomeopenres.16544.2 33644404 PMC7903200

[18] Schmidt AF Finan C Gordillo-Marañón M Asselbergs FW Freitag DF Patel RS. Genetic drug target validation using Mendelian randomisation. Nat Commun. (2020) 11:3255. doi: 10.1038/s41467-020-16969-0 32591531 PMC7320010

[19] Skrivankova VW Richmond RC Woolf BAR Yarmolinsky J Davies NM Swanson SA. Strengthening the reporting of observational studies in epidemiology using mendelian randomization: The STROBE-MR statement. JAMA. (2021) 326:1614–21. doi: 10.1001/jama.2021.18236 34698778

[20] Willer CJ Schmidt EM Sengupta S Peloso GM Gustafsson S Kanoni S. Discovery and refinement of loci associated with lipid levels. Nat Genet. (2013) 45:1274–83. doi: 10.1038/ng.2797 PMC3838666 24097068

[21] Borén J Taskinen M-R Björnson E Packard CJ. Metabolism of triglyceride-rich lipoproteins in health and dyslipidaemia. Nat Rev Cardiol. (2022) 19:577–92. doi: 10.1038/s41569-022-00676-y 35318466

[22] Ridker PM. LDL cholesterol: Controversies and future therapeutic directions. Lancet. (2014) 384:607–17. doi: 10.1016/S0140-6736(14)61009-6 25131980

[23] Ference BA Kastelein JJP Ray KK Ginsberg HN Chapman MJ Packard CJ. Association of triglyceride-lowering LPL variants and LDL-C-lowering LDLR variants with risk of coronary heart disease. JAMA. (2019) 321:364–73. doi: 10.1001/jama.2018.20045 PMC6439767 30694319

[24] Huang W Xiao J Ji J Chen L. Association of lipid-lowering drugs with COVID-19 outcomes from a mendelian randomization study. Elife. (2021) 10:e73873. doi: 10.7554/eLife.73873 34866576 PMC8709572

[25] Rosoff DB Bell AS Jung J Wagner J Mavromatis LA Lohoff FW. Mendelian randomization study of PCSK9 and HMG-coA reductase inhibition and cognitive function. J Am Coll Cardiol. (2022) 80:653–62. doi: 10.1016/j.jacc.2022.05.041 35953131

[26] Zhao SS Yiu ZZN Barton A Bowes J. Association of lipid-lowering drugs with risk of psoriasis: a mendelian randomization study. JAMA Dermatol. (2023). 159(3):275–80. doi: 10.1001/jamadermatol.2022.6051 PMC9878432 36696131

[27] Chang CC Chow CC Tellier LC Vattikuti S Purcell SM Lee JJ. Second-generation PLINK: rising to the challenge of larger and richer datasets. GigaSci. (2015) 4:7. doi: 10.1186/s13742-015-0047-8 PMC4342193 25722852

[28] 1000 Genomes Project Consortium Auton A Brooks LD Durbin RM Garrison EP Kang HM. A global reference for human genetic variation. Nature. (2015) 526:68–74. doi: 10.1038/nature15393 26432245 PMC4750478

[29] Teumer A Chaker L Groeneweg S Li Y Di Munno C Barbieri C. Genome-wide analyses identify a role for SLC17A4 and AADAT in thyroid hormone regulation. Nat Commun. (2018) 9:4455. doi: 10.1038/s41467-018-06356-1 30367059 PMC6203810

[30] Dönertaş HM Fabian DK Valenzuela MF Partridge L Thornton JM. Common genetic associations between age-related diseases. Nat Aging. (2021) 1:400–12. doi: 10.1038/s43587-021-00051-5 PMC7610725 33959723

[31] Bycroft C Freeman C Petkova D Band G Elliott LT Sharp K. The UK Biobank resource with deep phenotyping and genomic data. Nature. (2018) 562:203–9. doi: 10.1038/s41586-018-0579-z PMC6786975 30305743

[32] Hemani G Bowden J Davey Smith G. Evaluating the potential role of pleiotropy in Mendelian randomization studies. Hum Mol Genet. (2018) 27:R195–208. doi: 10.1093/hmg/ddy163 PMC6061876 29771313

[33] Emdin CA Khera AV Kathiresan S. Mendelian randomization. JAMA. (2017) 318:1925. doi: 10.1001/jama.2017.17219 29164242

[34] Haycock PC Burgess S Wade KH Bowden J Relton C Davey Smith G. Best (but oft-forgotten) practices: the design, analysis, and interpretation of Mendelian randomization studies. Am J Clin Nutr. (2016) 103:965–78. doi: 10.3945/ajcn.115.118216 PMC4807699 26961927

[35] Brion M-JA Shakhbazov K Visscher PM. Calculating statistical power in Mendelian randomization studies. Int J Epidemiol. (2013) 42:1497–501. doi: 10.1093/ije/dyt179 PMC3807619 24159078

[36] Nikpay M Goel A Won H-H Hall LM Willenborg C Kanoni S. A comprehensive 1,000 Genomes-based genome-wide association meta-analysis of coronary artery disease. Nat Genet. (2015) 47:1121–30. doi: 10.1038/ng.3396 PMC4589895 26343387

[37] Bowden J Davey Smith G Haycock PC Burgess S. Consistent estimation in mendelian randomization with some invalid instruments using a weighted median estimator. Genet Epidemiol. (2016) 40:304–14. doi: 10.1002/gepi.21965 PMC4849733 27061298

[38] Burgess S Bowden J Fall T Ingelsson E Thompson SG. Sensitivity analyses for robust causal inference from mendelian randomization analyses with multiple genetic variants. Epidemiology. (2017) 28:30–42. doi: 10.1097/EDE.0000000000000559 27749700 PMC5133381

[39] Hemani G Zheng J Elsworth B Wade KH Haberland V Baird D. The MR-Base platform supports systematic causal inference across the human phenome. Elife. (2018) 7:e34408. doi: 10.7554/eLife.34408 29846171 PMC5976434

[40] Giambartolomei C Vukcevic D SChadt EE Franke L Hingorani AD Wallace C. Bayesian test for colocalisation between pairs of genetic association studies using summary statistics. PloS Genet. (2014) 10:e1004383. doi: 10.1371/journal.pgen.1004383 24830394 PMC4022491

[41] UK Biobank. Neale lab . Available online at: http://www.nealelab.is/uk-biobank (Accessed August 17, 2023).

[42] Astle WJ Elding H Jiang T Allen D Ruklisa D Mann AL. The allelic landscape of human blood cell trait variation and links to common complex disease. Cell. (2016) 167:1415–1429.e19. doi: 10.1016/j.cell.2016.10.042 27863252 PMC5300907

[43] Ahola-Olli AV Würtz P Havulinna AS Aalto K Pitkänen N Lehtimäki T. Genome-wide association study identifies 27 loci influencing concentrations of circulating cytokines and growth factors. Am J Hum Genet. (2017) 100:40–50. doi: 10.1016/j.ajhg.2016.11.007 27989323 PMC5223028

[44] Lyon MS Andrews SJ Elsworth B Gaunt TR Hemani G Marcora E. The variant call format provides efficient and robust storage of GWAS summary statistics. Genome Biol. (2021) 22:32. doi: 10.1186/s13059-020-02248-0 33441155 PMC7805039

[45] Chen J Spracklen CN Marenne G Varshney A Corbin LJ Luan J. The trans-ancestral genomic architecture of glycemic traits. Nat Genet. (2021) 53:840–60. doi: 10.1038/s41588-021-00852-9 PMC7610958 34059833

[46] Dupuis J Langenberg C Prokopenko I Saxena R Soranzo N Jackson AU. New genetic loci implicated in fasting glucose homeostasis and their impact on type 2 diabetes risk. Nat Genet. (2010) 42:105–16. doi: 10.1038/ng.520 PMC3018764 20081858

[47] Carter AR Sanderson E Hammerton G Richmond RC Davey Smith G Heron J. Mendelian randomisation for mediation analysis: current methods and challenges for implementation. Eur J Epidemiol. (2021) 36:465–78. doi: 10.1007/s10654-021-00757-1 PMC8159796 33961203

[48] Wang Q Oliver-Williams C Raitakari OT Viikari J Lehtimäki T Kähönen M. Metabolic profiling of angiopoietin-like protein 3 and 4 inhibition: a drug-target Mendelian randomization analysis. Eur Heart J. (2021) 42:1160–9. doi: 10.1093/eurheartj/ehaa972 PMC7982288 33351885

[49] Lanzolla G Vannucchi G Ionni I Campi I Sileo F Lazzaroni E. Cholesterol serum levels and use of statins in graves’ Orbitopathy: A new starting point for the therapy. Front Endocrinol (Lausanne). (2019) 10:933. doi: 10.3389/fendo.2019.00933 32038490 PMC6987298

[50] Stein JD Childers D Gupta S Talwar N Nan B Lee BJ. Risk factors for developing thyroid-associated ophthalmopathy among individuals with graves disease. JAMA ophthalmology. (2015) 133(3):290–6. doi: 10.1001/jamaophthalmol.2014.5103 PMC4495733 25502604

[51] Nilsson A Tsoumani K Planck T. Statins decrease the risk of orbitopathy in newly diagnosed patients with graves disease. J Clin Endocrinol Metab. (2021) 106:1325–32. doi: 10.1210/clinem/dgab07 33560351

[52] Lanzolla G Sabini E Leo M Menconi F Rocchi R Sframeli A. Statins for Graves’ orbitopathy (STAGO): a phase 2, open-label, adaptive, single centre, randomised clinical trial. Lancet Diabetes Endocrinol. (2021) 9:733–42. doi: 10.1016/S2213-8587(21)00238-2 34592164

[53] McCarey DW McInnes IB Madhok R Hampson R Scherbakova O Ford I. Trial of Atorvastatin in Rheumatoid Arthritis (TARA): Double-blind, randomised placebo-controlled trial. Lancet. (2004) 363:2015–21. doi: 10.1016/S0140-6736(04)16449-0 15207950

[54] Stüve O Youssef S Steinman L Zamvil SS. Statins as potential therapeutic agents in neuroinflammatory disorders. Curr Opin Neurol. (2003) 16:393–401. doi: 10.1097/01.wco.0000073942.19076.d1 12858078

[55] Smith TJ Janssen JAMJL. Insulin-like growth factor-I receptor and thyroid-associated ophthalmopathy. Endocr Rev. (2019) 40:236–67. doi: 10.1210/er.2018-00066 PMC6338478 30215690

[56] Smith TJ Kahaly GJ Ezra DG Fleming JC Dailey RA Tang RA. Teprotumumab for thyroid-associated ophthalmopathy. N Engl J Med. (2017) 376:1748–61. doi: 10.1056/NEJMoa1614949 PMC5718164 28467880

[57] Bifulco M Ciaglia E. Statin reduces orbitopathy risk in patients with Graves’ disease by modulating apoptosis and autophagy activities. Endocrine. (2016) 53:649–50. doi: 10.1007/s12020-015-0762-z 26438397

[58] Ghavami S Yeganeh B Stelmack GL Kashani HH Sharma P Cunnington R. Apoptosis, autophagy and ER stress in mevalonate cascade inhibition-induced cell death of human atrial fibroblasts. Cell Death Dis. (2012) 3:e330–0. doi: 10.1038/cddis.2012.61 PMC3388233 22717585

[59] Li H Wang C-C Zhang M Li X-L Zhang P Yue L-T. Statin-modified dendritic cells regulate humoral immunity in experimental autoimmune myasthenia gravis. Mol Cell Neurosci. (2015) 68:284–92. doi: 10.1016/j.mcn.2015.08.010 26311508

[60] Kersten S. Angiopoietin-like 3 in lipoprotein metabolism. Nat Rev Endocrinol. (2017) 13:731–9. doi: 10.1038/nrendo.2017.119 28984319

[61] Lotta LA Stewart ID Sharp SJ Day FR Burgess S Luan J. Association of genetically enhanced lipoprotein lipase–mediated lipolysis and low-density lipoprotein cholesterol–lowering alleles with risk of coronary disease and type 2 diabetes. JAMA Cardiol. (2018) 3:957–66. doi: 10.1001/jamacardio.2018.2866 PMC6217943 30326043

